# Blood Culture Contamination Mitigation: Sustaining Success and Stewardship Systemwide

**DOI:** 10.1017/ash.2024.168

**Published:** 2024-09-16

**Authors:** Mark Povroznik

**Affiliations:** WVU Medicine

## Abstract

**Background:** False-positive blood cultures compromise care; extended stays, Clostridioides difficile risk increases and renal woes tagged to antibiotic alms contribute to the doubling of patient in-hospital mortality that is observed relative to true negative diagnostic results. False-positive affiliated central line-associated bloodstream infection reports can further obfuscate the quality of care provided. Between personnel performance pressures, laboratory resource losses and the risk for financial penalty under Value-Based Purchasing and Hospital-Acquired Condition Reduction Programs (Centers for Medicare & Medicaid Services), it becomes difficult to brush aside the burden of a non-zero blood culture contamination rate. Following unsuccessful and unsustainable endeavors that included educational exercise and waste tube employment, initial-specimen diversion devices designed to shift performance burdens from technicians to technology were co-opted in an effort to secure reliable blood culture contamination rate reductions. **Methods:** A 3.6% blood culture contamination rate was observed systemwide prior to intervention, which began in 2020. Among seventeen facilities that share a data system, twelve co-opted initial-specimen diversion device technology as the evidence-based anchor to a trifurcate intervention strategy that included value analysis and cultural curation. **Results:** The 2023 systemwide blood culture contamination rate was 1.95%; down from 2.8% in 2022 and 3.2% in 2021. The average cost per false-positive event was $2,111, with intervention amounting to systemwide savings of $4.1 million in 2023 as approximately 1,920 patients avoided false-positive incidents. Critical to year-over-year systemwide uptick in adoption of interventive technology was consistent and near real-time communication to caretakers regarding outcomes. **Conclusion:** The sustained success of the multifactorial solution showcased herein stems from the coupling of an evidence-based action with an ongoing assessment of value and communication channels carefully constructed to celebrate and perpetuate value observed. Layered uncertainties often cloud the crux of a multifactorial solution to a complex conundrum; for many decades the literature-supported solution to high blood culture contamination rates was to educate every person involved in every possible way. Only recently, following recommended practice revisions endorsed by the Clinical and Laboratory Standards Institute and Centers for Disease Control and Prevention, did it become apparent nationwide that education alone was insufficient; some contaminant pathways persist without meticulously mechanical closure. Antimicrobial stewardship requires the respectful removal of adaptable pressures from microorganisms, but the inverse is equally important; by setting an ambitious systemwide blood culture contamination rate target of 1% or less, it is hoped that all facilities involved herein respond to this pressure with optimism, introspection and innovation.

